# Supplementary material for: The Effect of Mineralocorticoid Receptor 3 Antagonists on Anti-Inflammatory and Anti-Fatty Acid Transport Profile in Patients with Heart Failure
Source: Cells. 2022 Apr 8;11(8):1264. doi: 10.3390/cells11081264 (PMC9027091; doi:10.3390/cells11081264)
Supplement: Supplementary file 1 [file cells-11-01264-s001.zip › cells-1646594-supplementary.pdf]

Supplementary Table S1. Sequence 5' --> 3' of primers forward and reverse

Primer	Sequence 5' --> 3'
ADIPOQ forward	TGGTGAGAAGGGTGAGAA
ADIPOQ reverse	AGATCTTGGTAAAGCGAATG
ACTB forward	TTCTGACCCATGCCCACCAT
ACTB reverse	ATGGATGATGATATCGCCGCGCTC
DEFA3 forward	TCCCAGAAGTGGTTGTTTCC
DEFA3 reverse	CAGAATGCCCAGAGTCTTCC
CD31 forward	AACAGTGTTGACATGAAGAGCC
CD31 reverse	TGTAAAACAGCACGTCATCCTT
a-SMA forward	CCGACCGAATGCAGAAG GA
a-SMA reverse	ACAGAGTATTTGCGCTCCGAA
FABP4 forward	TACTGGGCCAGGAATTTGAC
FABP4 reverse	GTGGAAGTGACGCCTTTCAT
CD11b forward	CAGCCTTTGACCTTATGTCATGG
CD11b reverse	CCTGTGCTGTAGTCGCACT
CXCR2 forward	CGAAGGACCGTCTACTCATC
CXCR2 reverse	AGTGTGCCCTGAAGAAGAGC
CD36 forward	TCTTTCCTGCAGCCCAATG
CD36 reverse	AGCCTCTGTTCCAACTGATAGTGA
COL1A2 forward	TCGCACATGCCGTGACTTG
COL1A2 reverse	GATAGCATCCATAGTGCATCCTTG
PREF1 forward	CTGGACGGTGGCCTCTATGAATG
PREF1 reverse	ATCATCCACGCAGGTGCCTC
CD68 forward	CTTTGGGCAAGGTTTCTCCTGCC
CD68 reverse	CTCCGGATGATGCAGAAAGC
CD3 forward	TGCTCCACGCTTTTGCCGGAGGACAG
CD3 reverse	TAGGAGGAGAACACCTGGACTACTC
ITLN1 forward	AACAGCTCCCTGCTGAGGTA
ITLN1 reverse	GCTGGCCATAGGGTGAGTAA
